# Infective Endocarditis

**Published:** 1903-11-21

**Authors:** Arthur Maude


### Hospital Clinics and Medical Progress.

### INFECTIVE ENDOCARDITIS.

By Arthur Maude, M.R.C.S.Eng., L.R.C.P.

This title is probably the least objectionable of the various names which have been applied to this condition (ulcerative endocarditis, malignant endocarditis, mycosis endocardiæ, vegetative endocarditis, etc.).

It has been long recognised that certain forms of endocarditis are septic in origin. Bouilland, in 1824, and Stenhouse Kirkes, in 1853, recognised clearly the pyæmic infection of the circulation, and Dr. Wilks, who suggested the name "arterial pyæmia," established this pathological view. Winge, in Sweden, first observed the presence of microbes on the affected valves—from his description, probably some variety of streptococcus. A very large body of bacteriological work was effected in this field between 1885 and 1890, but the range of inquiry is by no means closed yet.

The number of organisms now known [1] to produce the condition called infective endocarditis is already very large. We may classify them roughly into two groups :—

1. Cocci and bacilli known to exist in other infectious diseases. Streptococci, including that of erysipelas. Dr. F. Andrewes has also described a variety of streptococcus which he considers not to have been previously described. Staphylococci, viz., S. pyogenes aureus and S. pyogenes albus. The gonococcus. Pneumococcus of Fränkel, and the pneumobacillus of Friedlander. The typhoid bacillus, tubercle bacillus, and diphtheria bacillus. The B. coli communis, and bacilli of the paracolon group.

2. A series of organisms have been found (by Weichselbaum, Netter, Fränkel, Saenger, Gilbert, and Lion) not known to exist in other diseases ; to these the observers have attached various names : Bacillus endocarditis griseus ; micrococcus endocarditidis rugatus ; B. endocarditidis capsulatus ; B. immobilis fœtidus ; the bacillus of Gilbert and Lion ; and several other bacilli of less specific character.

It must be noted that all these are bacilli. In the last year Drs. Poynton and Paine [2] claim to have discovered a diplococcus which is the same as that found present in rheumatic fever. This organism we may for our purpose dismiss ; for, granted that there is a specific diplococcus found as the cause of rheumatic fever, its presence would remove a case of endocarditis into the classification of "rheumatic," in sharp contradistinction to "infective." The gist of Dr. Poynton's researches is to show that acute rheumatism is a specific infection produced by one form of diplococcus, whereas Senger and many others have held that rheumatism may be caused by a number of pyogenitic organisms. There is nothing remarkable in the fact that some cases of rheumatic endocarditis assume a "malignant" aspect—that is, tend towards ulceration or vegetative growth of valves. It is probable, we think, that ulceration of valves is gauged by the extent of the colonies of organisms (whatever their variety) which have landed on the valve, and that many vegetations are an attempt in the direction of repair.

In a large proportion of cases only one form of organism is present ; in the 25 cases so carefully investigated by Dr. Ware there was a mixed infection in only eight. It must also be borne in mind that even such an authority as Weichselbaum [3] has in undoubted cases been unable to detect any organism at all.

The influence of previous injury, or inflammatory damage, to the cardiac valves in the production of infective endocarditis has been from time to time discussed with varying estimates. The series of cases collected by the late Professor Kanthack [4] should, we think, render further discussion unnecessary. He found old cardiac lesions in 68 out of 84 cases. In one instance, a girl of 16, no evidence of previous endocarditis was found, but there was a patent foramen ovale, deficient interventricular septum, and pulmonary stenosis. Rupture of cardiac valves may be followed by infection even if there has been no previous valvular disease. An interesting point to be traced in Dr. Ware's paper is that the cases in which there was no evidence of old standing heart disease were all "secondary"—that is, they exhibited definite channels of infection, such as necrosis of bone, otitis media, pyosalpinx, pneumonia, &c. But of the cases in which endocarditis had existed for some time previously, very few showed any assignable channel of infection.

We must, however, remember that the point of entrance of the infective organism may be so small, so ill-defined, as to be overlooked or forgotten by the patient, and may present no trace afterwards even at a post-mortem examination. We do not believe that there are ever primary or idiopathic cases of the disease, but that all are the result of external infection. It is strange that until the meeting of the British Medical Association in 1900 [5] so little attention had been drawn to the wounds of dental operation as sources of infection. In addition to the instances cited at that meeting, the last two cases of malignant endocarditis in the writer's practice were debilitated anæmic servant-girls, the subjects of old mitral disease, in whom infective endocarditis followed the extraction of numerous fangs. When old valvular mischief exists, the liability to infective endocarditis is not lessened by full compensatory hypertrophy.

[1] Allbutt's Syst. Med.. vol. i., p. 626 ; St. Barth. Hosp. Rep, 1899, xxxv., p. 253. [2] Brit. Med. Jour., 1902, i., pp. 895–1028. [3] Ziegler and Nauwerck's Beiträge z. Path. Anat. und Allgem. Path., iv., 1889. [4] Ware, *loc. cit.* [5] Brit. Med. Jour., vol. ii., 1900.

*(To be continued.)*

